# Unveiling the therapeutic potential of the gut microbiota–brain axis: Novel insights and clinical applications in neurological disorders

**DOI:** 10.1097/MD.0000000000043542

**Published:** 2025-07-25

**Authors:** Okechukwu Paul-Chima Ugwu, Michael Ben Okon, Esther Ugo Alum, Chinyere Nneoma Ugwu, Emeka Godson Anyanwu, Basajja Mariam, Fabian Chukwudi Ogenyi, Val Hyginus Udoka Eze, Chinyere Nkemjika Anyanwu, Joseph Obiezu Chukwujekwu Ezeonwumelu, Simeon Ikechukwu Egba, Daniel Ejim Uti, Hope Onohuean, Patrick Maduabuchi Aja, Melvin Nnaemeka Ugwu

**Affiliations:** a Department of Publication and Extension, Kampala International University, Kansanga, Kampala, Uganda; b Department of Anatomy, Faculty of Biomedical Sciences, Kampala International University, Kansanga, Kampala, Uganda; c Health Care and Data Management, Leiden University, Leiden, Netherlands; d Department of Microbiology and Immunology, Kampala International University, Kansanga, Kampala, Uganda; e Faculty of Pharmacy, Kampala International University, Kansanga, Kampala, Uganda; f Biopharmaceutics Unit, Department of Pharmacology and Toxicology, School of Pharmacy, Kansanga, Kampala, Uganda; g Department of Biochemistry, Kampala International University, Kansanga, Kampala, Uganda; h Department of Medical Biochemistry, Faculty of Basic Medical Sciences, State University of Medical and Applied Science, Igbo Eno, Enugu, Nigeria.

**Keywords:** dysbiosis, fecal microbiota transplantation (FMT), gut microbiota–brain axis, neurological disorders, psychiatric disorders

## Abstract

Over the last several years, the gut microbiota–brain axis has been the focus of medical study, demonstrating the bidirectional nature of gut and brain communication and the resulting influence on neurological and mental health. Trillions of microorganisms, particularly those found in the gastrointestinal tract, contribute the most to the pathophysiology recovery of organs that are critical to human health, such as digestive processes and metabolism, immune responses, and even cognitive function. Dysbiosis (a disturbance in the microbiome balance) has been identified as one of the risk factors for neuropsychiatric illnesses such as depression, anxiety, autism spectrum disorder, Parkinson’s disease, and Alzheimer’s disease. Therapeutic strategies aimed at the gut microbiota, such as probiotics, dietary modifications, prebiotics, and fecal microbiota transplantation, will eventually offer ways to alleviate symptoms associated with these disorders by restoring microbial balance, modulating the immune response, and influencing the production of major neurotransmitters. Innovative drug carriers, such as microbially-derived nanoparticles and probiotics that target particular parts of the gut or microbial communities, may improve pharmaceutical treatment efficacy and specificity. The resolution of difficulties such as ethical concerns, unexpected repercussions, and peak performance optimization in a clinical setting is critical for the advancement of this subject.

## 1. Introduction

In recent years, scientists have been more interested in the gut microbiota–brain axis, which is concerned with how the gut microbiota and central nervous system (CNS) interact.^[1]^ This axis represents a bidirectional communication system that has a broad influence on many physiological processes and plays an important role in the pathophysiology of neuropsychiatric diseases.^[2]^ Understanding these mechanisms is vital for developing innovative ways and improving illness treatment.^[3]^ Trillions of microbes, comprising bacteria, viruses, fungi, and archaea, inhabit the human gastrointestinal tract (GIT), with the majority of them residing in the colon.^[4]^ This intricate and diverse microbial community, collectively termed the gut microbiota, performs a wide range of processes that are essential for human health, including proper digestion and metabolism, immune regulation, and even brain function.^[5]^ Diet, lifestyle, drugs, and genetic predispositions all have an impact on the makeup and diversity of the gut microbiota.^[6]^ Gut–brain connection occurs via numerous channels, including neuronal, immunological, endocrine, and metabolic signaling processes.^[7]^ The vagus nerve acts as the primary channel for this interaction, bringing messages back and forth between the gut and the CNS.^[8]^ Additionally, microbial metabolites, such as short-chain fatty acids (SCFAs) and neurotransmitters, regulate neuronal activity and the immune response, subsequently influencing brain function and behavior.^[9]^ Dysbiosis, a term developed for disruptions in the gut microbiota composition and functioning, has been linked in the development of numerous neurological and mental diseases such as depression, anxiety disorders, autism spectrum disorders (ASD), Parkinson’s disease (PD), and Alzheimer’s disease.^[10]^ Dysbiosis affects the natural balance of neurotransmitters, increases neuroinflammation, and undermines the integrity of the blood–brain barrier, exacerbating neurophysiological symptoms and causing the development of neurodegenerative illnesses.^[11]^ The new studies show that the use of microbiome modifying medicines may be a beneficial strategy for managing or possibly treating certain of the neurological illnesses.^[12]^ Probiotics, live microorganisms with health benefits, dietary adjustments, prebiotics, which foster the production of healthy gut bacteria, and faecal microbiota transplantation (FMT) found to be effective in reducing the symptoms connected to these conditions.^[13]^ These substances operate to restore microbial balance, mediate immunological responses, and influence neurotransmitter levels 13. Modern advancements in medication delivery methods give innovative avenues in regulating the gut microbiota–brain axis.^[13]^ Nanoparticles produced by microbial cells, microbiota-targeted probiotic formulations, microbiota-modulating hydrogels, and microbiota-responsive nanoparticles are seen as the most promising ways of delivering therapeutic agents, probiotics, prebiotics, or neuroactive compounds to specific locations in the gut or certain microbial communities.^[14]^ Despite significant progress in unlocking the therapeutic potential of the gut microbiota–brain axis, a number of obstacles remain. Top efforts should include addressing ethical issues about informed consent, monitoring of adverse consequences such as dysbiosis and antibiotic resistance, and governance in research and clinical practice.^[15]^ Furthermore, the understanding of the complicated mechanisms behind these medicines, as well as the design of their clinical success, are critical for the field’s progress.^[16]^ The gut–brain microbiota forms a complex and dynamic network that has a significant impact on neurological and mental health.^[17]^ Understanding the complex interplay between gut bacteria and the CNS provides tremendous prospects for the development of innovative therapeutic options that address unmet medical needs among patients with neurological illnesses.^[18]^

### 1.1. The aim of the review

The aim of this review is to look at the novel involvement of the gut microbiota–brain axis in neurological illnesses. This review explores the most recent studies on how gut microbiota impacts the brain in order to unearth novel strategies to cure disorders and use these insights in clinical practice. We reviewed the role of gut microbiota and the brain in the development and prevention of neurological diseases, as well as evaluated new treatments and delivery methods targeted at the gut microbiota for conditions such as Alzheimer’s disease, PD, and other neurodegenerative or psychiatric disorders.

## 2. Materials and methods

### 2.1. Search strategy

The search was carried out online using PubMed/MEDLINE, Scopus, Web of Science, and Google Scholar. To find papers in English, search phrases such as “gut microbiota–brain axis,” “neurological disorders,” “therapeutic interventions,” and “drug delivery systems” were employed. The literature search was conducted on items published between January 2010 and December 2024. The search initially produced 1450 items. After applying the inclusion and exclusion criteria, 145 studies were selected for evaluation.

### 2.2. Inclusion criteria

Only papers that matched the following criteria were considered for the review:

1. Investigate how bacteria in the gut and nerve system impact brain health, particularly in connection to neurological illnesses.
2. Investigate therapies that link the gut, brain, and microbiome, and explore novel medication delivery methods to change gut bacteria.
3. All research must be published in English between January 2010 and December 2024.

### 2.3. Exclusion criteria

The following criteria were used to exclude articles:

1. Flaws or biases in research might affect the accuracy of outcomes.
2. Studies unrelated to the microbiota gut–brain axis or neurological diseases.
3. Non-English writings or those published before or after the designated time period.

### 2.4. Data extraction and synthesis

The essential information from the research was acquired using a well-organized form:

- \tStudy design.
- \tSample size and population.
- \tInterventions (e.g., treatment and medication delivery).
- \tEffects on neurological diseases.
- Important findings.

The findings were presented by categorizing data and emphasizing patterns and gaps in previous research. The research were organized according to their key topics, such as gut–brain connection, innovative techniques to medication administration, and various therapy options.

### 2.5. Quality Assessment and critical analysis

We employed the scale for the assessment of narrative review articles to evaluate the quality of the narrative reviews in this research. The study’s design and methods needed to be reviewed.

Examples of prejudice include selection and reporting bias:

- Sample size and selection methods.
- Data analysis techniques.

According to these guidelines, all of the studies were classified as poor, moderate, or high in quality. When discrepancies were discovered, the studies were reviewed again by a different reviewer.

A detailed analysis was carried out to assess the quality of evidence, identify potential biases, and highlight the shortcomings of each research. The researchers looked at how well the data supported therapy and how it influenced how illnesses are seen.

### 2.6. Ethical considerations

All of the studies reviewed addressed participant anonymity, informed consent, and conflicts of interest. The research was conducted in accordance with relevant agencies’ ethical requirements, and no extra data were acquired for this study.

### 2.7. Review protocol

A set of guidelines was devised and adhered to throughout the evaluation process. This protocol included the search strategy, study selection guidelines, data extraction procedures, and methods for evaluating and combining research.

## 3. Role of gut microbiota in neurological disorders

The gut microbiota is altered in composition in neurodegenerative and neuropsychiatric diseases.^[19]^ Neurological disorders are conditions affecting the central and peripheral nervous systems that may injure the brain, spinal cord, cranial and peripheral nerves, autonomic nervous system, and neuromuscular muscles.^[20]^ Brain bleeding may be caused by a range of situations, including vascular diseases of blood vessels, difficulties that arise as a result of brain worries, backbone or brain injuries, and brain tumors.^[21]^ The gut microbiota in humans is strongly associated with the development of several nervous system disorders, including dysbiosis.^[22]^ In stark contrast, people with neurological symptoms and healthy controls had very different microbiota compositions.^[23]^ Importantly, communication between the gut microbiota and the brain varies throughout life, as evidenced by neurodevelopmental diseases (such as autism spectrum disorder), neurodegenerative diseases (such as PD and Alzheimer’s disease), and behavioral disorders (such as depression and anxiety).^[11]^ According to current animal and human (association) study, which were most likely changes in microbial diversity, such CNS alterations may have negative health implications and lead to anomalies (in the CNS) concerns ASD, depression, and anxiety.^[24]^ Communication between hosts and intestinal bacteria occurs via the synthesis of a great number of metabolites, including neurotransmitters such as gamma-aminobutyric (GABA), serotonin, dopamine (DA), and noradrenaline, as well as vitamins and SCFAs, almost exclusively within the gut.^[25]^ This method, however, may allow some of these substances to get across the blood–brain barrier, enter brain tissue, and impact the neuronal loops involved in addiction.^[26]^ SCFA, the major metabolite produced by colonic bacteria during the fermentation of dietary fiber, has a crucial role in altering neuroimmunoendocrine, metabolic homeostasis, infectious, and inflammatory function.^[27]^ SCFA is carefully managed as a helpful fuel for neurones and glial cells in the CNS, playing a vital role in brain development 28. SCFAs, such as butyrate and propionate, have been demonstrated in experiments to not only block histone deacetylase, but also to activate a particular subset of the host’s G protein-coupled receptors that are important for epigenome modification.^[9]^ Furthermore, symbiotic bacteria in the GIT contribute significantly to the creation and development of the host immune system.^[28]^ Similarly, the composition of metabolizing compounds in the gut microbiota, as well as molecular microbial patterns of the GIT (microbe-associated molecular patterns), activate immune cells, regulating synaptic information transmission and brain behavioral function.^[29]^ The study found that GI microbiota products had a significant influence on microglia activation both before birth and throughout adulthood.^[30]^ Furthermore, the microglia regulated by these products are critical to the proper functioning of the inflammatory process in the CNS. Microglia have been shown to have an important role in the correct synaptic wiring of the CNS during brain development.^[31]^ The gut microbiota controls the number and activity of microglial cells and their components, which causes human neurological disorders.^[32]^ Furthermore, germ-free syndrome in SCFA-treated mice demonstrates the complex pathway of microflora signaling, and it is worth noting that microglia function recovery in germ-free animals is reliant on the role of signals received from GI microflora.^[33]^ Along with clinical evidence of a possible relationship between gut disturbance and neurological disorders, scientists discovered a new dimension in the microbiota gut–brain circuit that affects many parts of the brain.^[34]^ While past research have revealed that a portion of autistic people suffer from persistent constipation, increased intestinal permeability, abdominal discomfort, and a disrupted intestinal microbiota, these findings suggest a relationship between dysbiosis and neurodevelopmental issues.^[35]^ Microbial transfer from the mother to the fetus, delivery mode, antibiotic use, and dietary intake may all have an effect on the fetus’s face and maturable colonization.^[36]^ These geographical factors alter the composition and function of intestinal commensals, which may have long-term ramifications for host health and contribute to future illnesses.^[37]^ Furthermore, it has been shown in mice that administering an antibiotic affects the flora of the mother’s and her child’s digestive tracts, resulting in decreased locomotor activity and behavioral changes in the neonates.^[38]^ Clinical studies demonstrate that antibiotic-induced dysbiosis is linked to the development of a number of neurodevelopmental diseases, including schizophrenia, depression, and bipolar illness.^[39]^ Thus, an excess of the colonizing microbiota following an early birth, known as dysbiosis, raises the baby’s risk of developing psychosis such as depression or schizophrenia.^[40]^ More study is required to discover the biological link between all of these features and people who are at risk of having mental disorders. Modulating the early-life microbiota might be a potential therapeutic for ASD and other neurological illnesses.^[41]^

## 4. Pharmacological targets in the gut microbiota–brain axis

The gut microbiota–brain axis is considered a bidirectional communicative system between the CNS and the gut microbiota, which are responsible for the regulation of the homeostasis and affect different psychological realities as well as some neurological and psychiatric disorders^[42]^ Attempting to impact this axis, biochemically is a plausible therapeutic intervention to handle a variety of neurological diseases.^[43]^ For instance, probiotic species like *Lactobacillus* and *Bifidobacterium* can be administered to alter gut microbiota, a therapy that may be beneficial in the management of mood disorders such as depression, anxiety, and autism.^[44]^ Some gut bacteria, on the other hand, can be able to produce neurotransmitters, for instance, serotonin as well as gamma-aminobutyric acid drugging such pathways may in effect alter the release and activity of neurotransmitters in the brain.^[45]^ The gut microbiota synthesized the SCFAs, such as acetate, propionate, and butyrate that are the key regulators of immune function, inflammation, and the wellness of the brain.^[9]^ Regulating SCFA production and activity may be of medicinal value in the course of some neurological disorders.^[46]^ Delivery of neurotrophic factors, like brain-derived neurotrophic factor (BDNF), and to boost their production relies on the gut microbiota, may facilitate neuronal survival, synaptic plasticity, and cognitive function in neurological disorders.^[47]^ Providing antioxidant support and minimizing oxidative stress with medicinal agents and modulating diverse actions of the gut microbiota could inhibit neuronal damage, prevent neurodegeneration, and support the brain health and function.^[48]^

## 5. Probiotics and prebiotics as emerging therapies

**Figure 1.**
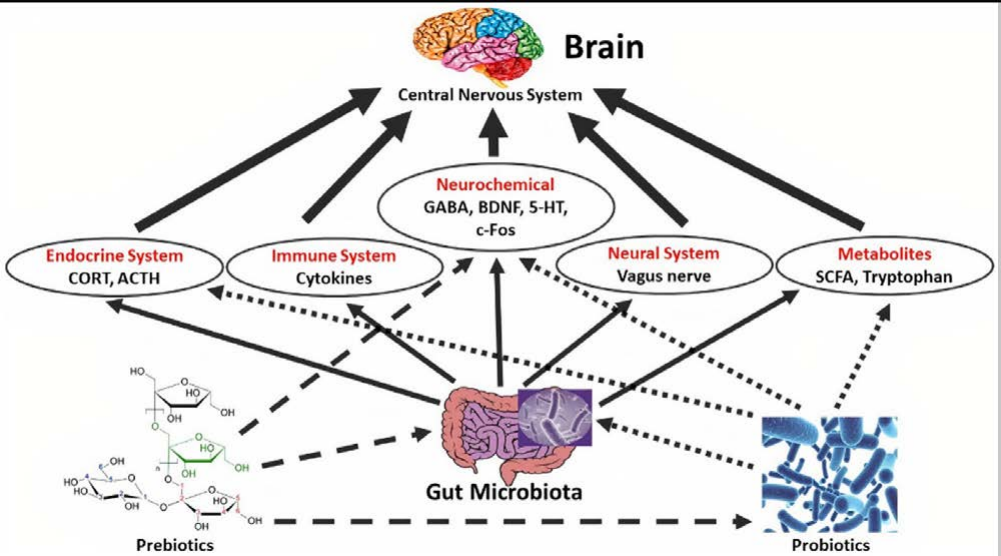
Showing the effect of probiotics on the central nervous system/CNS through the effect on the microbiota gut-brain axis. ACTH = adrenocorticotropic hormone, BDNF = brain-derived neurotrophic factor, c-Fos = cellular-Fos, CNS = central nervous system, CORT = corticosteroid, GABA = gamma-aminobutyric acid, 5-HT = 5-hydroxytryptamine, SCFA = short-chain fatty acid.

The researchers found differences in gut microbiota between healthy people and clinical patients with various neurologic diseases.^[49]^ The microbiome composition has been shown to respond to dietary interventions, altering the gut–brain axis activity.^[50]^ Many therapeutic strategies have been used to treat gut microbiome dysbiosis, which restores microbial balance in the intestines and improves clinical outcomes in neurological illnesses, including probiotics.^[51]^ The term “probiotic” was coined in 1974, and the World Health Organisation defines it as live microorganisms that have a positive influence on host health when taken in appropriate amounts.^[52]^ Prebiotics, on the other hand, are compounds produced from nondigestible dietary fibers that may particularly boost the formation and activity of good gut bacteria, leading to an increase in numerous studies have shown that gut microbe (GM) regulates the gut–brain axis, meaning that GM has an important role in the prevention and treatment of Alzheimer’s, depression, and insomnia.^[53]^ While pharmaceutical drugs remain vital in treatment, the popularity and utilization of microbial compounds known as probiotics and prebiotics, which are both safe and preventive, has expanded.^[54]^ Probiotics may positively alter the gut microbiota, and as a consequence, they have been recommended as a helpful tool for treating a variety of brain diseases.^[55]^ Furthermore, the presence of prebiotics has a direct impact by decreasing pathogenic bacteria colonization, altering the intestinal microbiota balance, and boosting the quantity of probiotics in the colon.^[56,57]^ This practice has the same therapeutic effect on mental illnesses as illustrated in Figure 1.

According to Figure 1, probiotics have both direct and indirect effects on brain function. Probiotic bacteria influence the hypothalamic-pituitary-adrenal (HPA) axis via altering corticosteroid and adrenocorticotropic hormone levels.^[58]^ The immune system is influenced by reduced pro-inflammatory cytokine production and inflammation, as well as stimulation in the CNS.^[59]^ Probiotics may also directly affect CNS biochemistry, such as via varying 5-hydroxytryptamine, BDNF, GABA, DA, and c-Fos levels, therefore influencing mind and behavior.^[59]^ The vagus and enteric nerves are also engaged in gut–brain communication and are influenced by some probiotic strains.^[58]^ Furthermore, probiotic microorganisms modulate the gut microbiota by increasing the range and composition of beneficial bacteria 63. At that stage, the gut microbiota may regulate metabolites such as SCFAs, exopolysaccharides, and tryptophan, which indirectly improves CNS function.^[60,61]^ Furthermore, the gut microbiota works with the immunological, endocrine, and nervous systems.^[58]^ The presence of probiotics alters and increases metabolites such as tryptophan and SCFAs, which directly affect brain function, and the secretion level of some brain factors such as GABA, serotonin/5-hydroxy tryptamine, BDNF, and DA, which ultimately affect mental disorders.^[62,63]^ Some probiotics impair the HPA tension feedback, which controls mood and emotion, resulting in reduced corticosteroid levels.^[64,65]^ Under the effect of probiotics and prebiotics, the immune system produces and secretes pro-inflammatory cytokines, which subsequently decrease inflammation in the target tissue, the brain, by altering the neurones and the hormone system.^[66,67]^ Studies have shown that the use of synbiotic products, which combine probiotics (e.g., *Lactobacillus*, *Enterococcus*, and *Bifidobacterium*) with prebiotics (e.g., resistant starch and inulin), produces a high level of neurotransmitters and neuropeptides, such as GABA and BDNF, improving CNS function and counting psychiatric disease-related functions such as anxiety, depression, stress, and memory ability.^[67]^

## 6. Psychobiotics

Psychobiotics refers to probiotics, prebiotics, and all microbiota-targeted interventions that can manipulate microbiota gut–brain signals and have positive effects on neurological functions such as mood, cognition, and anxiety.^[68]^ As it has been stated, depression and anxiety are disorders with high prevalence worldwide.^[69]^ Although there is a wide array of therapeutic options to treat them, undesirable secondary effects accompany most of them.^[70]^ Psychobiotics may regulate the neurotransmitters and proteins, including GABA, serotonin, glutamate, and BDNF, which play important roles in controlling the neural excitatory-inhibitory balance, mood, cognitive functions, learning, and memory processes.^[71]^

### 6.1. Psychobiotics mechanisms of action

**Figure 2.**
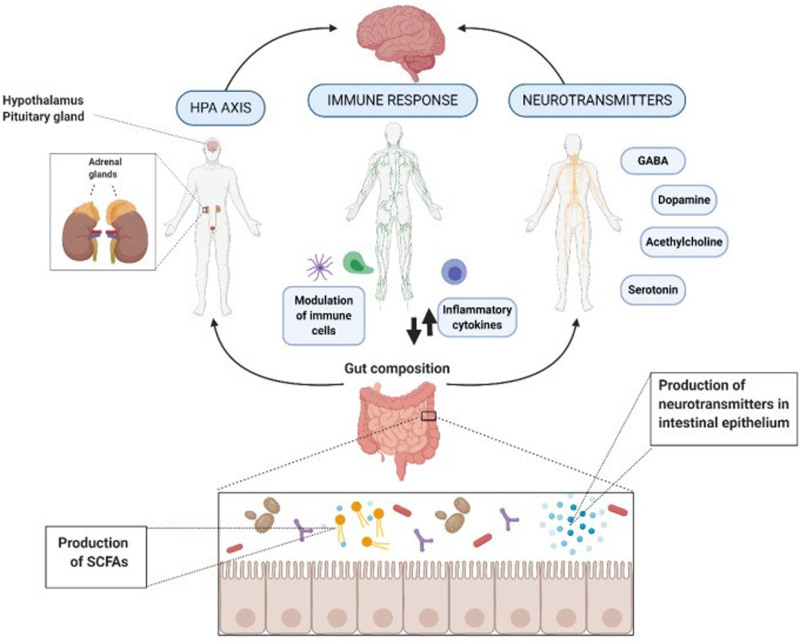
showing Action mechanisms by which the gut microbiota exerts the potential psychobiotic effect. GABA = gamma-aminobutyric acid, HPA = hypothalamic-pituitary-adrenal, SCFAs = short-chain fatty acids.

The investigation of the degree of complexity of the psychobiotic processes for which bacteria are utilized, although exclusively recognized, has not yet achieved complete detail.^[72]^ The argument is that bacteria exert their beneficial effects via the small intestine’s enteric nervous system or immune system activation.^[73]^ The following section discusses how psychological biofeedback improves psychophysiological indicators of sadness and anxiety.^[74]^ In reality, this may be accomplished by inhibiting the activity of the stress response system the HPA axis, for example or by lowering systemic inflammation.^[75]^ Furthermore, it may be achieved by combining direct immune system activation with the production of substances such as neurotransmitters, proteins, and short fatty acid chains.^[76]^ Figure 2 is useful for elucidating action pathways.

Using psychobiotics to treat neurological problems via the gut–brain connection is seen as a forward-thinking and novel method of treatment that can be depended on.^[77]^ Psychobiotics may intervene by exerting diverse effects on microbiota composition and activity, resulting in changes in neurotransmitter synthesis, immunological function, and metabolite production.^[78]^ These psychobiotic effects have a favorable influence on brain processes and enhance behavioral patterns.^[79]^ Neurodegenerative illnesses are still classified as having undiscovered causes; however, some variables such as lifestyle, food, aging, and heredity may contribute to the disease’s onset and progression.^[80]^ Prebiotics operate positively via the microbiome GIT balance to boost mental health.^[81]^ SCFAs, for example, predominantly exert their effects via immunological, endocrine, neurological, and humoral pathways.^[82]^ SCFAs have been discovered to penetrate melasa noc receptors for free fatty acids, which may disturb immune cells and intestinal epithelial cells, influencing the integrity and function of the intestinal mucosa.^[83]^ In this context, it also causes systemic inflammation and neuroinflammation via interleukin action and modulation of microglia cellular characteristics, as well as impairment of their functions.^[84]^ Furthermore, it increases the release of gastrointestinal hormones such as GLP-1, which are sent to the upper side via the vagus nerve and systemic networks.^[85]^ These channels are developed, which will ultimately have an influence on the brain’s learning, emotion, and memory.^[86]^ Alzheimer’s disease^[87]^ is one of the most common neurological disorders that cause dementia. According to studies undertaken by,^[88]^ these tau proteins are the primary causes of intracellular neurofibrillary tangles and the development of external amyloid plaques. This understanding of the disease’s physiological underpinnings will aid in the creation of the most effective treatments.^[89]^ Destroying the microbial bacteria in the stomach may cause cognitive deterioration associated with Alzheimer’s disease.^[90]^ Inflammation and oxidative stress harm neurones in the CNS, perhaps leading to Alzheimer’s disease.^[91]^ Finally, in order to reduce the cognitive consequences of d-galactose delivery by reactive oxygen species,^[92]^ cholinergic cell death must be inhibited and acetylcholine levels increased. Furthermore, overexposure to d-galactose intake for an extended period of time may result in diminished expression of brain nerve growth factors and related proteins.^[93]^ Nerve cell procedures were repeatedly weakened 96. Certain probiotic bacteria, most notably *Lactiplantibacillus plantarum*, may synthesize acetylcholine, protecting memory from a lack of d-galactose intake.^[94]^ PD is the second most common and main movement illness in the world.^[95]^ PD is primarily caused by synchlerin accumulation, as well as the progressive loss of motor symptoms such as tremor and stiffness caused by dopaminergic neuron depletion.^[95]^ The non-motor forms of the disorder are particularly accompanied by abnormalities in gut function, such as weight loss, gastroparesis, constipation, and defecation dysfunction.^[96]^ At the time, psychobiotic intake is the trendiest topic in PD treatment^[97,98]^ studied the effect of probiotic *Lacticaseibacillus paracasei* on motor dysfunction in PD rats and found that apomorphine rotation risk was lower than in saline-injected animals. Multiple sclerosis (MS) is an autoimmune illness characterized by the loss of myelin (a lipid coating) that supports axon fibers, resulting in their degradation in the CNS.^[99]^ The primary risk factors for MS include genetics and environmental factors, particularly viral infections.^[100]^ Nonetheless, the specific cause of this condition is not fully recognized by the scientific community.^[101]^ Over time, research have shown that probiotics help improve the immune system of people with MS by altering the microbiota in the gut, suppressing inflammatory pathways, and regulating the immunological system.^[102]^ ASD are a collection of social communication difficulties, unique sensory-motor behaviors, and intensely concentrated, limited interests that may have genetic or other common origins.^[103]^ ASD seems to be the same as it is in early infancy.^[104]^ It has been shown that people with autism are more likely to have GI problems. Gastrointestinal problems in autistic children is often associated with aggressive behaviors, sleep disorders, and hyperactivity.^[105]^ According to,^[106]^ probiotics reduce behavioral and gastrointestinal disturbances in ASD disorders. Probiotic combinations of *Lactobacillus acidophilus*, *Lacticaseibacillus rhamnosus*, and *Bifidobacterium longum* supplements given to autistic children for 3 months increased *Bifidobacteria* and *Lactobacilli* levels while also reducing weight and the appearance of GI issues. Out of all these mental health risks, the most prevalent is depressive disorder, followed by anxiety disorder.^[107]^ These 2 mental states most commonly coexist.^[108]^ Antidepressants, anxiolytics, and hypnotics are popular medications used to treat individuals.^[109]^ It has also been shown that the HPA axis temporarily malfunctions during periods of acute and chronic depression disorders.^[110,111]^ Studies have shown that the gut microbiota differs between normal persons and MDD patients.^[112]^ Changes in the gut microbiota include a decrease in *Bifidobacterium* and *Lactobacillus* and an increase in *Clostridium*, *Streptococcus*, *Klebsiella*, *Oscillibacter*, and *Allistipes*.^[113]^ Gut bacteria use vagal networks to traverse distress situations, according to the literature.^[114]^ After repeated stressor-related studies, norepinephrine is produced in a long-term manner, and this exposure alters the bacterial population and increases the permeability of colonic cells to bacteria and toxins, triggering another response on the HPA axis^[115,116]^ investigated the effect of the probiotic strain *Bifidobacterium breve* CCFM1025 on not only fecal micromolecules, but also cytokine and SCFA levels, neuronal changes, and corticosterone concentrations in the brain.^[117]^ Furthermore, a considerable impact was made to reducing sadness and anxiety-like behaviors.^[118]^

### 6.2. Psychobiotics and faecal microbiota transplantation (FMT)

#### 6.2.1. Psychobiotics and FMT

Psychobiotics, which are probiotics that may improve mental health by altering gut flora, are widely explored due to their potential to cure depression, anxiety, and cognitive decline.^[96]^ Studies have indicated that strains like *Lactobacillus* and *Bifidobacterium* might reduce stress and improve mood.^[97]^ Giving microorganisms from a healthy donor’s gut to a patient via FMT has helped cure irritable bowel syndrome (IBS) and PD.^[97]^

#### 6.2.2. Possible harm and concerns

Psychobiotics and FMT may be beneficial, although they have certain hazards.^[96]^ According to certain clinical research, individuals may have symptoms such as gastrointestinal pain, bloating, and mild infections.^[98]^ Furthermore, it is unclear how safe psychobiotics may be for the most fragile persons.^[99]^ FMT may be harmful since it can transmit germs and compromise the immune system.^[99]^ Some patients who had FMT suffered serious infections, including those caused by multidrug-resistant organisms, highlighting the need of thorough screening and monitoring.^[100]^ The study is restricted in several aspects and sometimes yields contradictory findings.^[101]^ The study on psychobiotics and FMT is still in its early phases, since many studies are tiny, do not use the same methodology, and have inconsistent findings.^[102]^ Many of these studies were conducted over a short period of time, so we don’t know much about the long-term impacts or how long the advantages persist.^[103]^ Sometimes studies show that neurological problems may not improve significantly or at all.^[103]^ For example, some studies have suggested that psychobiotics may assist with mood disorders, while others were unable to corroborate these findings or showed very minor benefits, raising concerns about the reliability of the data.^[104]^ FMT research may provide conflicting outcomes because to differences in donor microorganisms, procedures employed, and recipient characteristics.^[101]^ As a result, many people continue to dispute how successfully FMT heals neurological illnesses, and further well-planned experiments are needed to precisely define its applications.^[102]^

There are significant ethical concerns about psychobiotic and FMT therapy. There is concern regarding informed consent for psychobiotics, particularly among patients with mental health issues, who may be unaware of the dangers and benefits.^[103]^ Concerns have also been raised concerning the economic aspect of psychobiotics, as well as the danger of exploiting patients’ conditions.^[104]^ It is still contentious to provide psychobiotics to youngsters, pregnant women, and the elderly since their safety has not been thoroughly investigated.^[105]^ FMT sparks further ethical questions, most notably over donor selection, the use of unscreened microorganisms, and the risk of transmitting hazardous bacteria to the recipient.^[106]^ It is also critical to get donor agreement, since some may not see microbiota donation as a gift and may be unaware of the hazards associated.^[106]^ Measures to control FMT, such as screening, monitoring, and alerting about potential hazards, are still being developed and should be addressed immediately.^[107]^ Another difficulty is that FMT might be seen as either a medical intervention or an organ donation, which raises ethical concerns when used.^[107]^

#### 6.2.3. Drug delivery systems

The exploration of innovative drug delivery methods directed at regulatory molecules in the gut microbiota for therapeutic applications is a rapidly developing field that encompasses the most revolutionary scientific achievements.^[119]^ These creative approaches are set to advance the effectiveness, specificity, and safety of therapeutic interventions through the targeted delivery of drugs, microorganisms, prebiotics, or other bioactive compounds to the microbiome of the gut or to the gut–brain axis of stress and anxiety.^[120]^ Here’s an overview of some novel drug delivery systems designed to target the gut microbiota for neurological interventions as shown in Table 1.

**Table 1:** Different interventions of drug delivery systems.

Drug delivery system	Description	Therapeutic application	Potential benefits	Challenges/limitations
Microbial-derived nanoparticles	Nanoparticles derived from microorganisms (e.g., OMVs or bacterial ghosts) used to integrate pharmaceuticals and deliver them to targeted gut areas. These nanoparticles help with the absorption and transport of nanomedicine, boosting its effectiveness in reaching target locations	Transport probiotics, prebiotics, psychobiotics, or neuroactive substances to modulate microbiota composition and activity for neurological treatments	Improved targeting and efficacy of microbiome-based therapeutics through encapsulation, minimizing degradation and ensuring precise delivery	Difficulty in ensuring precise targeting and avoiding off target effects. Potential issues with degradation and stability of nanoparticles
Microbiota-targeted probiotic formulations	Genetically engineered or selected probiotics designed to survive harsh digestive conditions and actively alter gut microbiota composition in specific areas. These probiotics can produce or deliver neuroactive substances like neurotransmitters to modulate the gut–brain axis	Alter gut microbiota composition to enhance neurotransmitter generation, decrease inflammation, and improve immune response for treating neurological conditions	Ability to deliver neuroactive compounds to the gut–brain axis, improving mental health outcomes and reducing inflammation	Survival and effectiveness of probiotics in harsh gastrointestinal environments. Variability in therapeutic outcomes due to individual microbiota differences
Microbiota-modulating hydrogels	Hydrogels designed to deliver probiotics, prebiotics, or other agents to the gut in a controlled manner. They can be customized with microbial nutrients to promote beneficial bacteria such as *Bifidobacteria* and *Lactobacilli*, aiding in gut microbiota composition and function	Deliver probiotics or prebiotics to the gut in a controlled way, modulating microbiota for therapeutic effects and reducing side effects	Controlled release of agents, potentially reducing side effects and increasing therapeutic efficacy in gut microbiota manipulation	Complexity in designing hydrogels with the right balance of stability, biodegradability, and specific microbial nutrient content
Microbiota-responsive nanoparticles	Nanoparticles engineered to remain indigestible until reaching specific areas of the gut. These nanoparticles disintegrate in response to bacterial enzymes, pH levels, or metabolites, releasing their payload to modulate microbiota composition and treat neurological disorders	Modulate the gut microbiome by delivering therapeutic agents to specific gut regions affected by dysbiosis or neurological abnormalities	Specific targeting of areas affected by dysbiosis or neurological issues, leading to precise modulation of microbiota composition	Ensuring nanoparticles are stable and effective in different microbiome environments. Risk of unintended effects from premature payload release
Fecal microbiota transplantation (FMT) pills	FMT capsules provide a noninvasive alternative to traditional FMT, encapsulating healthy donor fecal material to be transferred directly to the lower gastrointestinal tract. This treatment aims to restore healthy gut microbiota to treat neurological disorders like Parkinson’s disease, multiple sclerosis, and autism spectrum disorders	Restores gut microbiota composition to treat neurological diseases like Parkinson’s disease, multiple sclerosis, and autism spectrum disorders	Noninvasive treatment option for neurological diseases with potential for widespread clinical application	FMT capsule development is still in the early stages, with challenges in ensuring consistent efficacy and patient acceptance

FMT = fecal microbiota transplantation, OMVs = outer membrane vesicles.

#### 6.2.4. Microbial-derived nanoparticles

It has been shown that nanoparticles derived from microorganisms, such as outer membrane vesicles or bacterial ghosts, may be used to integrate pharmaceuticals by encapsulating them for targeted distribution to certain gut areas or the gut microbiota.^[120]^ Nanoparticles, which operate as anti-degradation barriers, may transfer microbiota straight into tissue and help in the absorption and transport of nanomedicine, boosting its chances of reaching its target location.^[120]^ Nanoparticles derived from bacteria may transport probiotics, prebiotics, putative psychobiotics, or neuroactive substances in the gut to modulate microbiota composition and activity for neurological treatment.^[121]^

#### 6.2.5. Microbiota-targeted probiotic formulations

New and innovative probiotic applications may be developed that can effectively pass through the acidic and harsh conditions of the digestive system and actively fill specific parts of the gut where the composition and activity of the gut microbiota can be favorably altered.^[122]^ These genetically engineered or chosen probiotics may be beneficial in the production or delivery of neuroactive substances, such as neurotransmitters that circulate the gut–brain axis.^[123]^ Probiotics derived from the microbiota that share characteristics with the brain maintain appropriate neurotransmitter generation, decrease inflammation, and improve immunological response.^[124]^

#### 6.2.6. Microbiota-modulating hydrogels

Hydrogels may be designed to preserve and convey content delivery agents, probiotics, or prebiotics to the gut in a controlled way.^[125]^ Microbiota-modulating hydrogels can be customized by adding specific microbial nutrients or modulators, thereby directing the development and activity of bacteria beneficial to the gut microbiota, such as *Bifidobacteria* and *Lactobacilli*, for the composition and function of the gut microbiota.^[126]^ Microbiota-altering hydrogels may be a promising route for tissue-specific medication delivery to the gut microbiota and gut–brain axis, boosting therapeutic efficacy while decreasing side effects.^[127]^

#### 6.2.7. Microbiota-responsive nanoparticles

The particle size of the nanoparticles may be adjusted such that they stay indigestible until they reach the area of the gut where specific changes in the microbiota composition or metabolic activity occur.^[128]^ These nanoparticles may be programmed to disintegrate or release the payload in the presence of certain bacterial enzymes, a predetermined pH level, or even specific metabolites that indicate dysbiosis or neurological abnormalities.^[57]^ These nanoparticles function by influencing gut microbiomes, allowing them to transport stabilized medicinal compounds to a specific location, modulating the makeup and activity of the gut microbiota and thereby treating neurological diseases.^[129]^

#### 6.2.8. Fecal microbiota transplantation (FMT) pills

FMT capsules are a novel form of rectal trend that replaces traditional invasive FMT treatments to provide patients with a healthy gut microbiota.^[130]^ FMT capsules may effectively transfer microbiota straight to the lower GIT by encapsulating healthy donors’ fecal material in the capsule lining, which protects it from stomach acidity.^[131]^ FMT tablets are being studied as a potential treatment for neurological illnesses such as PD, MS, and ASD.^[11,132]^ This is because these medications affect the makeup and activity of the gut bacteria.

#### 6.2.9. Clinical trials and evidence

One of the most notable discoveries has been the link between the gut microbiota and the brain axis for neurohormonal health and disease, which has resulted in an increase in clinical trials looking for pharmacological treatments targeting the gut microbiota to treat neurological conditions.^[133]^ There are many clinical studies looking at the usefulness of probiotics in lowering depression and anxiety by altering gut microbiota makeup and activity.^[78,134]^ In a randomised controlled trials (RCT) study, the combination of *Lactobacillus helveticus* and *B longum* probiotics was shown to be beneficial when administered to individuals suffering from depression. They provided a greater decrease in depressed symptoms than placebo,^[24,135]^ investigated if consuming *Lactobacillus casei* strain Shirota may reduce anxiety and stress levels in healthy persons, and discovered that probiotic users had considerably lower anxiety ratings and cortisol levels than those who were given placebos. New study has connected prebiotics to cognitive support, including the ability to reverse dementia by altering the gut microbiota and boosting the synthesis of physiologically beneficial metabolites.^[136]^ The clinical results of an RCT conducted in elderly people with mild cognitive impairment revealed that taking a specific type of prebiotic formulation outperformed the placebo, and this medicine recently improved cognitive function, as well as increasing the levels of valuable gut bacteria.^[137]^ A preclinical investigation conducted on a mouse model of Alzheimer’s disease found that food supplementation with prebiotic fiber improved memory and eradicated beta-amyloid buildup in the brain.^[138]^ On the other hand, it was reported that FMT is being investigated as a therapy for PD using gut microbiota-modulating chemicals.^[139]^ According to the research released by,^[126]^ a pilot study investigating the effects of FMT from healthy donors on PD patients discovered that the therapy resulted in improvements in motor issues, gastrointestinal disorders, and gut microbiota composition when compared to the baseline.^[140]^ Finally, a pilot trial found that a combined therapy of FMT and antibiotics reduced motor symptoms in PD while maintaining inflammation levels normal when compared to standard care.^[141]^ Other researchers are presently looking at psychobiotics, which are bacteria that live in the gut and produce neuroactive substances, as possible therapeutic agents for ASD by targeting the gut–brain axis.^[142]^ A research conducted by^[143]^ discovered that a specific probiotic strain was more helpful than a placebo in alleviating gastrointestinal symptoms, behavioral issues, and poor social communication skills in children with autism spectrum condition.^[142]^ According to an ASD research, psychobiotic preparations that regulate the development of the microbial population in the stomach treat ASD symptoms by increasing neurotransmitter production.^[142]^

#### 6.2.10. Challenges and future directions

A fresh, challenging, and, at the same time, some sort of integrated manufacturing course that fundamentally follows the gut–brain axis places us into an improved health concept.^[136]^ Finally, recent research presents a strong cognitive and genetic rationale for molecular manipulation of the microbial gut as a therapy for brain diseases.^[138]^ As qualitative treatment advances, families and individuals will encounter specific challenges.^[139]^ However, the underlying basis of the gut–brain axis for gut bacteria’s participation in brain functioning and behavior is unclear.^[140]^ They are complex, intrinsic networks. Making exact microbiological or lipid footprints very challenging.^[140]^ Therapy modules vary according to the diversity of the microbiota.

#### 6.2.11. Ethical consideration

The possibility to change the gut microbiota for the treatment of brain illnesses has raised significant ethical concerns that must be carefully considered and addressed. These ethical difficulties will continue to emerge in this industry.^[135]^ These issues must be addressed in order to ensure responsible and ethical behavior in research and clinical practice.^[136]^ Take into consideration that patients must be fully informed about the possible dangers, benefits, and uncertainties associated with therapeutic microbiota alteration before giving their agreement, and consent should be voluntary.^[139,140]^ Manipulating the gut microbiota might have unforeseen consequences, including as dysbiosis, antibiotic resistance, and systemic side effects; these must be monitored and controlled, and the negative effects of microbiome manipulation.^[141,142]^

### 6.3. *Stages of evidence in gut–brain axis research*

#### 6.3.1. Preclinical evidence

Preclinical evidence is often defined as research conducted in animal models or cell cultures to investigate the basic biology of the gut microbiota–brain axis and its role in neurological illnesses.^[143]^ These publications provide a conceptual framework for understanding how the gut microbiome affects brain health.^[143]^ Preclinical trials are important for identifying the specific bacteria species or metabolites that regulate brain functioning.^[144]^ They also look at how these microbial variables interact with the immune system, neurotransmitter pathways, and inflammation, as well as how they contribute to illnesses like anxiety, depression, and neurodegenerative disorders.^[145]^ Manipulation of the gut microbiota has been shown to impact behavior, cognitive ability, and neuroinflammation in animal models (germ-free or probiotic-fed mice).^[146]^ These findings are critical for understanding the potential therapeutic effect of microbiome-based treatments for neurological disorders.^[147]^ Despite the preclinical data, the results of animal research may not always match with those in people due to species differences in microbiome makeup, brain anatomy, and immune responses.^[147]^ Thus, preclinical data is just the beginning of the hunt for novel medicines and processes.

#### 6.3.2. Pilot clinical evidence

Pilot clinical trials are tiny studies conducted in humans to assess the feasibility, safety, and early effectiveness of therapies based on preclinical data.^[148]^ These studies often include fewer than 100 people and lack the statistical power to prove a convincing therapeutic benefit of gut microbiota modification.^[149]^ Pilot clinical studies can determine whether microbial therapies, such as probiotics or FMT, may effectively improve brain function in people.^[150]^ They will also be able to assess the side effects, tolerance, and dose-response relationships of various microbiome therapies.^[151]^ The studies focus on the effectiveness of microbial therapy in treating neurological conditions including depression, ASD, and PD.^[151]^ They also provide the first evidence on the impact of microbial therapies on brain-derived indicators such as cognitive performance, neuroinflammation, and mood regulation.^[67]^ Pilot clinical studies are often restricted in scope due to a small sample size, lack of control groups, and short follow-up periods, making it unable to make definite assertions about the effectiveness or long-term safety of microbiome-based therapeutics.^[152]^ The results are usually exploratory in nature and are used to guide more thorough investigations.^[152]^

#### 6.3.3. Large randomised controlled trials (RCTs)

Large RCTs with diverse participant populations are the gold standard in clinical research.^[153]^ Such trials are randomized, with participants assigned to 1 of 2 groups (e.g., treatment arm, receiving microbiome modulation therapy; or control arm, placebo), and it is possible to draw strong, statistically significant conclusions about whether a specific intervention is effective or safe.^[154]^

Large RCTs have provided solid evidence that microbiome-based therapeutics are both effective and safe.^[155]^ For example, they can authoritatively indicate if probiotics, prebiotics, or FMT have therapeutic benefit in neurological disorders, such as improving cognitive function in Alzheimer’s disease or decreasing anxiety in people with major depressive disorder.^[156]^ Large RCTs may provide valuable information on the processes by which microbiome therapies affect brain health by measuring clinical outcomes (e.g., changes in depression ratings, cognitive function, or motor symptoms in neurodegenerative illnesses).^[157]^ Such studies will also be able to assess how changes in the gut microbiota might be translated into measurable neurological advantages, such as inflammation reduction, neurotransmitter increase, or behavioral amelioration.^[157]^ Large RCTs are regarded the gold standard, yet they may be resource-intensive and time-consuming.^[153]^ The most typical issues they face include participant loss, patient variability in a research population, and the length of follow-up required to assess the long-term effect of therapies.^[158]^ Furthermore, certain outside variables, such as nutrition or environment, may influence the findings, making interpretation more challenging.^[158]^

#### 6.3.4. *Overcoming limitations and controversies of gut–brain therapies*

Research into gut–brain treatments as a technique of treating neurological illnesses has increased in recent years, due to a growing body of data linking gut bacteria to brain wellbeing.^[159]^ Nonetheless, despite the hopeful outcomes, these therapies have a number of limits and contentious issues that need much debate before they can be used safely and efficiently in clinical practice as shown in Table 2.^[160]^

**Table 2:** Overcoming limitations and controversies of gut–brain therapies.

Limitation/controversy	Description	Controversial aspect	Recommendation for overcoming the issue
Individual microbiota variability	The human gut microbiota is highly diverse and individualized, making it difficult to develop standardized microbiome therapeutics. Probiotics and prebiotics may work for some but not others due to baseline microbiota differences	Skeptics argue microbiome therapies may be overhyped as solutions for conditions like depression and neurodegeneration. The lack of personalized treatments may limit efficacy and lead to disappointing outcomes	Focus on personalized microbiome medicine to tailor treatments to individual microbiota profiles. Improve understanding of how baseline microbiota affects therapeutic efficacy
Inconsistent data and causality deficit	Existing studies on the gut–brain axis are mostly correlational, lacking causality. While a link between the microbiota and neurological disorders exists, it’s unclear if microbiome treatments can directly influence brain function	The speculative nature of current microbiome research and the lack of mechanistic clarity make it difficult to assess whether gut–brain therapies can address the underlying causes of neurological diseases or just alleviate symptoms	Future studies should focus on establishing causal links between microbiota and neurological diseases. Emphasis should be placed on controlled, mechanistic studies rather than observational data
Safety concerns with FMT	Microbiome treatments, particularly FMT, carry risks of pathogen transmission and unintended microbial changes. The long-term safety of FMT in treating neurological conditions is still under investigation	Ethical concerns around FMT involve the introduction of foreign microbiota into the human body, especially in neurodegenerative diseases. There are also worries about the lack of standardized donor screening and the potential for microbial imbalances or pathogen transmission	Develop and enforce rigorous safety protocols for FMT, including standardized donor screening, and long-term follow-up studies to monitor safety in neurological treatment
Limited treatment procedures	Microbiome therapies lack standardization in terms of probiotic strains, doses, and treatment durations. Variations in administration routes and forms (oral, rectal, capsules, etc) can affect treatment efficacy	There is ongoing debate whether the lack of standardization in microbiome therapeutics is a research bottleneck or a reflection of the microbiome’s inherent complexity. Some argue microbiome therapies should be personalized rather than 1-size-fits-all solutions	Standardize treatment protocols for microbiome therapies, including specific strains, dosages, and administration routes. Encourage more robust clinical trial designs to determine optimal treatment regimens
Ethical and regulatory issues	Regulatory standards for microbiome-based treatments, particularly those using genetically modified microbes, are underdeveloped. Agencies like the FDA have yet to create clear standards for these treatments, making their clinical application uncertain	The genetic modification of microbes for neuroactive substance production raises ethical questions about unintended consequences like immune system disturbances or the development of new diseases. Informed consent for microbiome treatments, particularly FMT, remains a concern	Establish clear regulatory guidelines for microbiome-based treatments, including genetically modified organisms, to ensure safety and efficacy. Implement regulatory oversight for FMT and other microbiome-modulating therapies
Durability and longevity of treatments	Gut–brain therapies have limited long-term efficacy, as many studies have short follow-up periods. The microbiome may revert to its original composition, raising doubts about the sustainability of therapeutic benefits	Experts argue that long-term studies are necessary to fully understand the risks and benefits of microbiome-based therapies. However, the cost and time required for these studies have delayed their implementation, and there is debate over whether these therapies provide long-term benefits or only temporary relief	Conduct long-term studies on microbiome therapies to determine sustainability of their effects. Investigate the potential for maintaining microbiome changes over time and ensuring long-term neurological benefits
Commercialization and profitability concerns	The commercialization of microbiome-based therapies faces financial challenges, particularly for unregulated products. Probiotics, prebiotics, and FMT are often marketed with exaggerated claims, and their safety and efficacy are not always well-supported by high-quality research	Scientists warn that the commercialization of microbiome therapies, particularly unregulated products like probiotics, may lead to patients receiving ineffective or dangerous treatments, raising ethical concerns about profit-driven research	Address concerns over commercialization by establishing ethical standards for the sale and marketing of microbiome-based therapies. Ensure transparency and evidence-based claims in product offerings

FDA = Food and Drug Administration, FMT = fecal microbiota transplantation.

##### 6.3.4.1. Individual microbiota variability limitations

The human gut microbiota is very diverse, with unique combinations influenced by nutrition, environment, genetics, and lifestyle. The variation makes it difficult to develop standardized microbiome therapeutics. Probiotics and prebiotics, for example, may assist 1 person but not another due to differences in the baseline microbiota.^[160,161]^

###### 6.3.4.1.1. Controversial aspect

Skeptics argue that microbiome-based therapies are at danger of being overhyped as a solution for illnesses including depression, anxiety, and neurodegeneration.^[162]^ The lack of individualization in these therapies may limit their efficacy and result in disappointing clinical outcomes. Many academics have expressed Skepticism about personalized microbiome treatment, wondering if we can grasp and regulate the complexity of individual microbiomes.^[162]^

##### 6.3.4.2. Inconsistent data and causality deficit

Despite increasing data linking gut microbiota to brain health, the causal relationship remains unclear.^[42]^ The majority of existing research are correlational, which means they indicate links between microbiome makeup and neurological disorders but do not establish cause-effect linkages.^[42]^ This precludes us from reaching a strong judgment about whether microbial therapies (e.g., probiotics, prebiotics, or FMT) may directly modify brain function in a therapeutically significant way.^[163]^

###### 6.3.4.2.1. Problematic aspect

It is problematic because current microbiome research is based on speculative results that are not mechanistically clear.^[162]^ The idea that the gut microbiota may interact directly with the brain is also controversial, since it is contested whether microbial treatments offer promise in treating neurological illnesses or whether they will just cure symptoms rather than addressing the underlying causes^[42]^ safety concerns. Microbiome treatments, particularly FMT, provide a risk of pathogen transmission and unintended microbial changes.^[163]^ Despite FMT’s encouraging outcomes in the treatment of gastrointestinal ailments such as Clostridium difficile infection, its use in neurological conditions is still experimental, and its long-term safety has not been well demonstrated.^[163]^

###### 6.3.4.2.2. Controversial aspect

The ethical implications of FMT are significant. Critics also question the safety of introducing alien microbiota into the human body, particularly in the case of neurodegenerative illnesses, when the long-term implications are unknown.^[162]^ Furthermore, the lack of standardized donor screening protocols increases the danger of microbial imbalances or the introduction of antibiotic-resistant germs, which may lead to major health problems.^[42,163]^

##### 6.3.4.3. Limited treatment procedures

Current microbiome-based therapeutics, including probiotics, prebiotics, and FMT, lack standardized procedures.^[164]^ Probiotic strains, dose regimens, and treatment durations are not properly specified across trials, resulting in inconsistent therapeutic effects. Furthermore, the mode of administration (oral, rectal, etc) and form of these drugs (e.g., capsules, powders) may alter the efficacy of these treatments.^[165]^

###### 6.3.4.3.1. Controversial aspect

The scientific community discusses whether the lack of standardization in microbiome therapeutics is a research bottleneck or a reflection of the microbiome’s complexity.^[164]^ Others contend that microbiome therapies should be more personalized rather than uniform, 1-size-fits-all solutions, which goes against the idea of universal treatments that apply to all individuals with neurological illness.^[165]^

##### 6.3.4.4. Limitations

Ethical and regulatory issues. Microbiome-based treatments, especially those using genetically modified microbes (e.g., psychobiotics) or FMT, are presently controlled in an immature manner.^[42]^ Regulatory organizations such as the FDA have yet to create thorough standards for the approval and commercialization of these medicines, making their clinical applicability questionable.^[42]^

###### Controversial feature

The genetic modification of microbes to synthesize neuroactive chemicals raises ethical problems.^[164]^ It is argued whether manipulating the microbiome would have unexpected consequences, such as uncontrolled bacterial proliferation, immune system disturbance, or even the development of new diseases.^[165]^ Furthermore, the problem of informed consent for patients undergoing microbiome treatments, namely FMT, is a source of worry, particularly given the unknown hazards of donor microbiota.^[165]^

##### 6.3.4.5. Durability and longevity

Gut–brain treatments have little long-term efficacy. Many studies, especially those using probiotics and FMT, have short follow-up periods (weeks to months), making it impossible to assess if the therapeutic benefits have been sustained.^[166]^ Changes in the microbiome may be temporary, and the original microbial profile can be recovered after a specific period of time, casting doubt on the long-term effectiveness of these therapies.^[166]^ Controversial Point: According to some experts, long-term research are required to fully understand the dangers and advantages of microbiome-based therapeutics. However, the cost and time required to conduct these studies have hampered their implementation.^[167]^ The idea that microbiome treatment might be used to treat chronic neurological conditions is still debated, with some arguing that such therapies may only provide a temporary fix to symptoms rather than a long-term, long-lasting remedy.^[168]^

##### 6.3.4.6. Questions about commercialization and profitability

The commercialization of microbiome-based medicines will be financially challenging, particularly for unregulated goods.^[169]^ The probiotics, prebiotics, and FMT markets are growing, but not without hazards.^[170]^ Most goods are advertised with exaggerated promises, and their efficacy and safety are not always supported by high-quality scientific research.^[171]^

###### 6.3.4.6.1. Controversial aspect

Some scientists warn against commercializing the microbiome, particularly probiotic supplements and microbiome-modulating drugs. Uncontrolled use of microbiome therapies may lead to patients receiving inadequate or even dangerous interventions, raising ethical concerns about financially driven research and commercialization.^[171]^

#### 6.3.5. Disorders affected by brain-gut axis disturbances and therapeutic options

Disruptions of the brain-gut axis are linked to a variety of illnesses and treatments as shown in Table 3.^[151]^ The gut–brain axis has been studied extensively for its role in a variety of neurological illnesses, with dysbiosis of the gut microbiota facilitating disease development and affecting brain function.^[151]^ Some neurological illnesses caused by brain-gut axis dysfunction and its potential therapy are listed below:

**Table 3:** Disorders affected by brain-gut axis disturbances and therapeutic options.

Disorder	Brain-gut axis connection	Therapeutic options	Potential impact of therapy
Depression	Intestinal microbial dysbiosis alters brain chemistry, especially neurotransmitter production like serotonin. Gut microbiota modulates mood regulation	Probiotics (*Lactobacillus*, *Bifidobacterium*), **psychobiotics**, **antidepressants (SSRI, SNRI)** that regulate mood and reduce inflammation	Improved **neurotransmitter production**, mood regulation, and reduced **neuroinflammation**
Anxiety disorders	Dysbiosis affects the **HPA axis**, increasing anxiety and stress. Gut microbiota imbalance influences the body’s stress response and emotional regulation	Probiotics, **cognitive behavioral therapy (CBT)**, **gut-targeted treatments** that modulate the gut–brain axis and reduce anxiety	Modulated stress response, reduced anxiety, and improvement in emotional regulation
Irritable bowel syndrome (IBS)	IBS is a gut–brain disorder where microbial imbalances contribute to both gastrointestinal and neurological symptoms like **brain fog** and discomfort	Probiotics, **prebiotics**, **FMT**, and **cognitive therapy** that alleviate both gastrointestinal discomfort and brain-related symptoms like brain fog	Improved gastrointestinal function and reduced **brain fog**, enhanced gut–brain communication
Parkinson’s disease	Gut microbiota disruption may trigger **neuroinflammation**, worsening motor symptoms and cognitive decline, particularly in **Parkinson’s disease**	Probiotics, **FMT**, **dopaminergic therapy**, and **gut-directed medications** that improve both motor function and gut health	Reduced **neuroinflammation**, improved **motor function** and cognitive performance in **Parkinson’s disease**
Alzheimer’s disease	Gut dysbiosis in Alzheimer’s may lead to **inflammation** and **amyloid plaque** formation, contributing to cognitive impairment and neurodegeneration	Gut-directed medications, **anti-inflammatory agents**, and **probiotics** that modulate gut inflammation and cognitive decline	Reduction in **neuroinflammation** and **amyloid plaque** buildup, potentially slowing cognitive decline
Autism spectrum disorder (ASD)	Dysbiosis in children with ASD has been linked to neurodevelopmental delays and behavioral problems, with potential effects on the brain’s development	Probiotics, **FMT**, **nutritional therapies**, and **psychological interventions** that improve gut health and behavioral symptoms	Improved gut–brain communication, alleviated **neurodevelopmental** and **behavioral** issues
Multiple sclerosis (MS)	In MS, gut microbiota imbalance can exacerbate **immune dysfunction**, increasing **neuroinflammation** and contributing to disease progression	Immunomodulatory treatments, **probiotics**, and **anti-inflammatory agents** that balance immune response and improve gut health in MS	Reduced **neuroinflammation**, regulation of immune responses, and improved gut microbiota balance
Wilson disease (neurodegenerative)	Wilson disease, a genetic disorder, involves **copper accumulation**, which can alter gut microbiota composition, contributing to neurodegeneration and cognitive symptoms	Chelation therapy (*penicillamine*), **zinc supplements**, **probiotics**, and experimental **FMT** to regulate gut microbiota and manage cognitive and behavioral symptoms	Regulated copper levels, improved **cognitive function**, and **neurodegenerative** symptom management

ASD = autism spectrum disorder, CBT = cognitive behavioral therapy, FMT = fecal microbiota transplantation, HPA = hypothalamic-pituitary-adrenal, IBS = irritable bowel syndrome, MS = multiple sclerosis, SNRI = serotonin–norepinephrine reuptake inhibitor, SSRI = selective serotonin reuptake inhibitor. * and ** indicates levels of statistical significance.

##### 6.3.5.1. Depression

Intestinal microbial dysbiosis may alter brain chemistry, particularly the creation of neurotransmitters such as serotonin. Gut microbiota may be altered using a range of therapeutic approaches, including probiotics (e.g., *Lactobacillus* and *Bifidobacterium*), psychobiotics, and antidepressants (SSRI, SNRI) that improve mood regulation.^[67]^

##### 6.3.5.2. Anxiety disorders

Dysbiosis of the gut microbiota affects the HPA axis, increasing anxiety and stress reactions. Probiotics, cognitive behavioral therapy, and gut-targeted treatments may all help to control the gut–brain communication channels that contribute to anxiety.^[152]^

##### 6.3.5.3. Irritable bowel syndrome (IBS)

IBS is a gut–brain disorder in which microbial imbalances cause gastrointestinal and neurological system symptoms. Probiotics, prebiotics, FMT, and cognitive therapy may help relieve gastrointestinal discomfort and improve brain fog caused by IBS.^[153]^

##### 6.3.5.4. Parkinson disease

Gut microbiota disruption may contribute to neuroinflammation in PD, worsening motor symptoms and cognitive loss. Probiotics, FMT, dopaminergic therapy, and gut-directed medicines are among treatments that may improve patients’ gut health and motor function.^[154]^

##### 6.3.5.5. Alzheimer’s disease

A dysregulated gut microbiota may lead to increased inflammation and amyloid plaque formation, both of which can contribute to cognitive impairment in Alzheimer’s. Gut-directed medicines, anti-inflammatory medications, and probiotics may help to modulate brain activity and halt the disease’s course.^[155]^

##### 6.3.5.6. Autism spectrum disorder (ASD)

Gut microbiota dysbiosis has been related to neurodevelopmental delays and behavioral problems in children with ASD. Probiotics, FMT, nutritional, and psychological therapies may all help to enhance gut and behavioral health.^[156]^

##### 6.3.5.7. Multiple sclerosis (MS)

Dysregulated gut microbiota has the potential to exacerbate the immunological dysfunction seen in MS, worsening neuroinflammation. Immunomodulatory treatments, probiotics, and anti-inflammatory substances may work together to regulate immune responses and the gut flora.^[157]^

##### 6.3.5.8. Wilson disease (neurodegenerative)

In Wilson disease, a hereditary disorder that causes copper buildup in the body, dysregulations in the gut microbiota may exacerbate neurodegeneration and contribute to cognitive and behavioral symptoms. Chelation treatment (e.g., penicillamine), zinc supplements, and probiotics might all be utilized to treat the disease’s gastrointestinal and neurological symptoms. FMT is experimental, but it might help regulate gut bacteria and improve symptoms.^[158]^ Microbiome-directed therapies have a high potential to become an effective tool in the treatment of neurological diseases, including Wilson disease, because microbiome-directed therapies, when used in conjunction with conventional options, may provide an opportunity to manage symptoms and improve patient outcomes.^[160,165,168]^ More research is needed to determine the long-term efficacy of these medicines for controlling chronic neurological illnesses.

## 7. Conclusion

Investigation of the microbiota-gut axis has notably deepened the awareness of the complicated interplay between the gut microorganisms and neuro health. The bidirectional communication (between the gut and the CNS) highlights the fundamental role of the GMs in regulating physiological processes and influencing the pathogenesis of several neurological and psychiatric disorders. Microbial therapeutics, such as probiotics, prebiotics, and FMT, which aim at restoring microbial balance, modulate the immune responses, and affect the neurotransmitter production are potential intervention which may help the patients manage symptoms by alleviating them. Moreover, innovative drug delivery systems such as microbial-derived nanoparticles and microbiota-targeted formulations potentially render the therapeutic process more effective and precise. However, being able to solve issues like ethical considerations, unintended impacts, and getting the maximum benefits out of treatment in clinical settings is of the paramount importance. In the next years, future research directions should pay attention to decoding mechanisms of gut–brain communication and designing personalized treatment strategies based on the unique character of given individual’s microbiota. Generalizing, the microbiota–brain axis is a developing and fascinating field of research with amazing impacts on the administration of and treatment of neurological conditions. The prospect of further evolution in this field has the ability to help in changing the methods of treatment and outcomes of the people affected by these incurable conditions as well.

## Author contributions

**Conceptualization:** Okechukwu Paul-Chima Ugwu.

**Data curation:** Michael Ben Okon, Esther Ugo Alum, Basajja Mariam.

**Formal analysis:** Michael Ben Okon, Esther Ugo Alum.

**Funding acquisition:** Esther Ugo Alum.

**Investigation:** Okechukwu Paul-Chima Ugwu, Michael Ben Okon, Esther Ugo Alum.

**Methodology:** Michael Ben Okon, Emeka Godson Anyanwu, Val Hyginus Udoka Eze.

**Resources:** Esther Ugo Alum.

**Software:** Simeon Ikechukwu Egba.

**Supervision:** Okechukwu Paul-Chima Ugwu, Esther Ugo Alum, Chinyere Nneoma Ugwu, Emeka Godson Anyanwu, Joseph Obiezu Chukwujekwu Ezeonwumelu, Simeon Ikechukwu Egba, Daniel Ejim Uti, Hope Onohuean, Patrick Maduabuchi Aja, Melvin Nnaemeka Ugwu.

**Validation:** Chinyere Nneoma Ugwu, Basajja Mariam, Fabian Chukwudi Ogenyi, Val Hyginus Udoka Eze, Chinyere Nkemjika Anyanwu, Joseph Obiezu Chukwujekwu Ezeonwumelu, Simeon Ikechukwu Egba, Daniel Ejim Uti, Hope Onohuean, Patrick Maduabuchi Aja.

**Visualization:** Chinyere Nneoma Ugwu, Basajja Mariam, Fabian Chukwudi Ogenyi, Val Hyginus Udoka Eze, Chinyere Nkemjika Anyanwu, Joseph Obiezu Chukwujekwu Ezeonwumelu, Simeon Ikechukwu Egba, Daniel Ejim Uti, Hope Onohuean, Patrick Maduabuchi Aja, Melvin Nnaemeka Ugwu.

**Writing – original draft:** Michael Ben Okon, Chinyere Nneoma Ugwu, Emeka Godson Anyanwu, Basajja Mariam, Fabian Chukwudi Ogenyi, Val Hyginus Udoka Eze, Chinyere Nkemjika Anyanwu, Joseph Obiezu Chukwujekwu Ezeonwumelu, Simeon Ikechukwu Egba, Daniel Ejim Uti, Hope Onohuean, Patrick Maduabuchi Aja, Melvin Nnaemeka Ugwu.

**Writing – review & editing:** Okechukwu Paul-Chima Ugwu, Esther Ugo Alum, Chinyere Nneoma Ugwu, Emeka Godson Anyanwu, Basajja Mariam, Fabian Chukwudi Ogenyi, Val Hyginus Udoka Eze, Chinyere Nkemjika Anyanwu, Joseph Obiezu Chukwujekwu Ezeonwumelu, Simeon Ikechukwu Egba, Daniel Ejim Uti, Hope Onohuean, Patrick Maduabuchi Aja, Melvin Nnaemeka Ugwu.
